# Partial splenic embolization to permit continuation of systemic chemotherapy

**DOI:** 10.1002/cam4.856

**Published:** 2016-09-09

**Authors:** Jose Hugo M. Luz, Paula M. Luz, Edson Marchiori, Leonardo A. Rodrigues, Hugo R. Gouveia, Henrique S. Martin, Igor M. Faria, Roberto R. Souza, Roberto de Almeida Gil, Alexandre de M. Palladino, Karina B. Pimenta, Henrique S. de Souza

**Affiliations:** ^1^ Department of Interventional RadiologyRadiology DivisionNational Cancer Institute, INCARio de JaneiroBrazil; ^2^ National Institute of Infectious Disease Evandro ChagasOswaldo Cruz FoundationRio de JaneiroBrazil; ^3^ Department of RadiologyFederal University of Rio de JaneiroRio de JaneiroBrazil; ^4^ Department of Clinical OncologyNational Cancer Institute, INCARio de JaneiroBrazil; ^5^ Department of AnesthesiologyNational Cancer Institute, INCARio de JaneiroBrazil

**Keywords:** Cancer, interventional Radiology, oxaliplatin, partial splenic embolization, spleen, systemic chemotherapy, thrombocytopenia

## Abstract

Systemic chemotherapy treatments, commonly those that comprise oxaliplatin, have been linked to the appearance of distinctive liver lesions that evolves to portal hypertension, spleen enlargement, platelets sequestration, and thrombocytopenia. This outcome can interrupt treatment or force dosage reduction, decreasing efficiency of cancer therapy. We conducted a prospective phase II study for the evaluation of partial splenic embolization in patients with thrombocytopenia that impeded systemic chemotherapy continuation. From August 2014 through July 2015, 33 patients underwent partial splenic embolization to increase platelets count and allow their return to treatment. Primary endpoint was the accomplishment of a thrombocyte level superior to 130 × 10^9^/L and the secondary endpoints were the return to chemotherapy and toxicity. Partial splenic embolization was done 36 times in 33 patients. All patients presented gastrointestinal cancer and colorectal malignancy was the commonest primary site. An average of 6.4 cycles of chemotherapy was done before splenic embolization and the most common regimen was Folfox. Mean platelet count prior to embolization was 69 × 10^9^/L. A total of 94% of patients achieved primary endpoint. All patients in need reinitiated treatment and median time to chemotherapy return was 14 days. No grade 3 or above adverse events were identified. Aiming for a 50% to 70% infarction area may be sufficient to achieve success without the complications associated with more extensive infarction. Combined with the better safety profile, partial splenic embolization is an excellent option in the management of thrombocytopenia, enabling the resumption of systemic chemotherapy with minimal procedure‐related morbidity.

## Introduction

More than 30 years ago, partial splenic embolization (PSE) started being used for two frequent consequences of increased portal vein pressure: esophageal variceal hemorrhage and platelet sequestration in spleen 1. Even though it revealed its value, it took a while for splenic embolization to get more accepted due to an elevated rate of adverse events, mainly spleen abscess formation, sepsis, spleen disruption, pancreas inflammation due to nontarget embolization and severe pulmonary infection 2. Recent literature, however, has demonstrated significant reduction in complication and mortality rates resulting from various reasons including improvements in the available materials for transcatheter embolization and angiography equipment, added to the changes in the PSE procedure, learned throughout the past 20 years 3, 4, 5, 6.

Currently, interventional radiology approach to the splenic artery is used to handle a myriad of diseases, such as splenic contusion, platelets sequestration, splenic artery aneurysmatic formations, increased portal vein pressure, and tumors of the spleen 2. It is also indicated in the management of reduced splenic artery flow, increasing blood irrigation to livers after hepatic transplantation and improving platelet counts in patients with thrombocytopenia in the course of anti‐cancer treatment or suppressive therapy in hepatitis C 3.

A decrease in hematological parameters, mainly platelets count drop, derived from splenic sequestration can appear in numerous medical conditions, frequently resultant of increased portal vein pressure and augmentation of spleen in patients with cirrhosis or those who present with disturbances of the main portal vein and its tributaries 7, 8. The reduction in the platelets count in the hypersplenism clinical picture is largely triggered through augmented splenic retention or thrombocytes damage, even though other implicated issues have been described such as thrombopoietin degradation by platelets retained inside the spleen. Reduction in the production of thrombopoietin in cases of extensive liver metastasis or advanced hepatic cirrhosis might likewise induce thrombocytopenia 9.

Systemic chemotherapy (SC), principally those based in oxaliplatin administration, induced liver toxicity and damage can provoke a medical state equivalent to hypersplenism present in patients with hepatic cirrhosis 10. Analysis of the normal liver after major hepatectomies in patients with colon and rectal cancer have already demonstrated distinguished toxic consequences on liver parenchyma derived from SC administration. When spleen enlargement and platelets sequestration develop in the course of the SC treatment, the consequential low platelets count can interrupt patient regimen or force dosage reduction, decreasing the efficiency of cancer therapy 11. Prescription attenuation or chemotherapy suspension is imposed when thrombocytopenia reaches 75 × 10^9^/L throughout cancer regimens administration. Chemotherapy‐induced thrombocytopenia (CIT) has been previously defined as an amount fewer than 75 × 10^9^/L that produces an inappropriate anti‐cancer therapeutic effect 12, 13.

## Methods

We formulated a prospective, phase II, single‐arm and single‐center study for the evaluation of PSE in patients with CIT that impeded SC continuation. From August 2014 through July 2015, 33 patients underwent PSE in order to increase platelets count to allow their return to SC. Spleen sequestration was the supposed origin of the CIT in our patients supported by medical history evaluation and laboratorial and radiological appraisal 10, 14. Our primary endpoint of interest was the accomplishment of a thrombocyte level superior to 130 × 10^9^/L and the secondary endpoints were the return to SC and the occurrence of adverse events. PSE was performed if the patient presented thrombocytopenia of 75 × 10^9^/L or less and after a consensus decision between the Clinical Oncology Department and the Interventional Radiology Team. Platelets counts were recorded prior to and 1, 2, 3, and 4 weeks after PSE. Pre‐ and post procedure computed tomography (CT) imaging was performed in all patients for the evaluation of splenic augmentation and infarction area. Before PSE, splenic dimensions were recorded in a single measurement fashion since it correlates well with total volume of the spleen and the finding of splenomegaly 11. To estimate necrosis, the same measurement routine was done, in the one to 2 week post‐PSE CT, taking into account the infarcted area and the preserved parenchyma. If a single measurement was not possible at the post‐PSE CT, because of its necrotic pattern appearance, a subjective analysis of the infarcted splenic parenchyma amount was done in agreement by two radiologists. Laboratory values were recorded before, during, and after the embolization procedures. Length of hospital permanence, SC regimen used prior to PSE, length of SC interruption, and the occurrence of adverse events were additionally recorded. CT of the superior abdomen was done 1 week after PSE and compared to the final post PSE angiography to evaluate splenic infarcted area accordance level. We opted not to give vaccinations since with partial splenic embolization patient's spleen immune protection would not be completely eliminated.

### PSE technique

**Figure 1:**
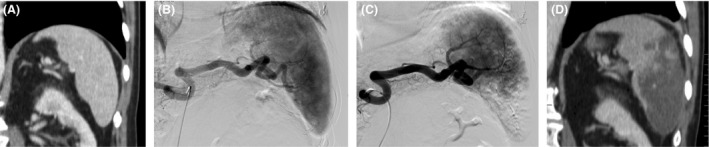
(A) Computed tomography showing enlarged spleen in a patient with colorectal cancer after 7 cycles of Folfox. (B) Pre‐embolization arteriogram showing usual splenic arterial anatomy. (C) Immediate post partial splenic embolization (PSE) arteriogram showing embolization of approximately 50% of the splenic parenchyma (D) Computed tomography 2 weeks after PSE showing partial spleen necrosis, which accurately correlated with the post PSE arteriogram.

PSE's were executed by one interventional radiology fellow and one staff member. Patients were sedated with intravenous medications. Dedicated pre, per, and post angiography was done to identify left gastroepiploic artery, short gastric branches and pancreatic body and tail branches in order to avoid unintended embolization 15. Following microcatheterization of branches supplying the splenic lower pole, embolization was executed with 1 vial of 100–300 microspheres (Bead Block® Terumo) mixed with 80 mg of gentamycin. We preferentially occluded splenic artery lower pole branches in order to minimize the occurrence of pleural effusion or subdiaphragmatic abscess 16. The superselective catheterization and the use of microspheres aims to achieve a more predictable embolized area as possible. Additional embolic material was used at the Interventional Radiologist discretion and endpoint was stasis for all procedures. For the embolized splenic parenchyma estimation, a final arteriogram was routinely performed. The embolization target for this study was to attain at least 50% of infarcted spleen tissue and to not go beyond 70% (Fig. 1) and this goal was estimated by the performing interventional radiologist at the end of every procedure. Subsequently, this endpoint analysis was confronted with the opinion of other interventional radiologist for agreement and in the case of divergence, a mean value was adopted. All patients received intravenous analgesia with 2 g of Dipirona during PSE and intravenous morphine (Dimorph®) at the operator depiction. Patients' immediate recuperation after PSE occurred in our interventional radiology beds for half dozen hours. We attempted, whenever possible, same‐day hospital discharged, but symptomatic patients were kept overnight for intravenous analgesia and antiemetic medications. All patients were oriented to take oral pain medication for 5 days and antibiotics for 7 days after PSE.

### Statistical analysis

Summary statistics were calculated for platelet count as well as other demographic and clinical characteristics of interest. Percent of patients achieving primary endpoint by week was calculated. The association of the binary primary endpoint of interest (thrombocyte level superior to 130 × 10^9^/L, yes/no) with clinical factors of interest was tested using logistic regression models. Additionally, the association between observed thrombocyte count at week 2 and clinical factors was tested using linear regression models. The clinical factors of interest included number of cycles, estimated fraction of spleen necrosis by angiography, estimated fraction of spleen necrosis by CT, type of material and size of particle. The correlation of spleen necrosis fraction by angiography with spleen necrosis fraction by CT was also tested using linear regression.

### Ethics approval, conflicts of interest, and funding

The ethics committee of the Brazilian's National Cancer Institute (INCA) approved the study protocol (Approval #31770814.7.000.5274). All patients provided written informed consent. There was no funding for this work. The authors declare no conflicts of interest.

## Results

PSE was done 36 times in 33 patients, 13 men and 20 women. The youngest patient was 32 years old and the oldest one 83 years old (mean age 58.8 years, median 59 years) and performance status ranged from 0 to 1. Gastrointestinal cancer was present in the absolute majority of the patients and colorectal cancer (*n* = 29 patients) was the most common primary site. Twenty patients presented hepatic metastases, while metastases were also present in the lung and peritoneum in 11 and four patients, respectively. An average of 6.4 cycles of SC was done before PSE and the most common chemotherapy regimen was Folfox, used in 24 patients (71%). Five patients received Xeloda (15%), two patients received Folfiri (6%), one patient received Folfirinox (3%), one patient received Sorafenib (3%), and one patient received 5FU alone (3%). Overall, 29 patients (88%) received some form of oxaliplatin‐based SC regimen. Two patients had hepatitis C and cirrhosis and one patient also had lymphoma.

**Figure 2:**
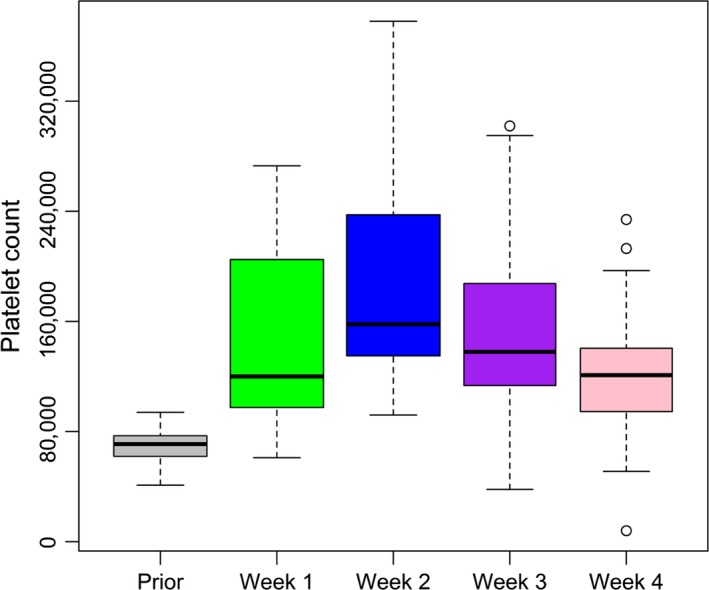
Platelets by week: Mean platelet count prior to partial splenic embolization (PSE) was 69 × 109/L. Mean platelet count at weeks 1, 2, 3, and 4 post‐PSE were, respectively, 141 × 109/L, 188 × 109/L, 154 × 109/L, 122 × 109/L. Platelets count new decrease at week 3 followed the return of patients to Systemic chemotherapy (SC) treatment.

All patients were submitted to PSE only once except for two patients: in one, PSE was performed twice and in another, it was performed three times due to subsequent declines in platelets count after SC was reinitiated. Mean platelet count prior to PSE was 69 × 10^9^/L, median of 71 × 10^9^/L, interquartile range 62–76 × 10^9^/L, maximum platelet count was 94 × 10^9^/L confirming low platelet count for all patients prior to PSE. Overall, 94% of patients achieved our primary endpoint. By week, the percent of patients achieving the primary endpoint was 39%, 86%, 63%, and 46% in weeks 1, 2, 3, and 4, respectively. Mean platelet count at weeks 1, 2, 3, and 4 post‐PSE were 141 × 10^9^/L (range 61–273 × 10^9^/L), 188 × 10^9^/L (range 92–378 × 10^9^/L), 154 × 10^9^/L (range 38–302 × 10^9^/L), 122 × 10^9^/L (range 8–234 × 10^9^/L), respectively. All patients in need (31 patients) were able to reinitiate treatment, and the median time to return to SC was 14 days (mean 17.7 days). Platelets count new decrease, starting at the third week, was accompanied by the return of patients to the SC treatment showing the direct relation of this novel decay Figure 2.

No grade 3 or above adverse events were identified. The most worrisome adverse event was an overnight upper gastrointestinal bleeding after PSE with no hemodynamic instability. This patient was kept overnight and underwent endoscopy that demonstrated gastric lacerations probably caused by forceful vomiting (Mallory–Weiss syndrome) with no need for any intervention. A CT was also performed showing, besides the embolized spleen, normal findings. This patient did not show a relevant decrease in the hemoglobin status and was discharged within 24 h. As for other adverse events, 12 patients (35%) referred low to moderate pain (three patients [9%] had severe pain), seven patients (21%) had low‐grade fever, seven patients (21%) had nauseas (two of which had vomiting). There were no significant changes in bilirubin: mean total bilirubin was 0.80 prior to and 0.59 after PSE.

**Figure 3:**
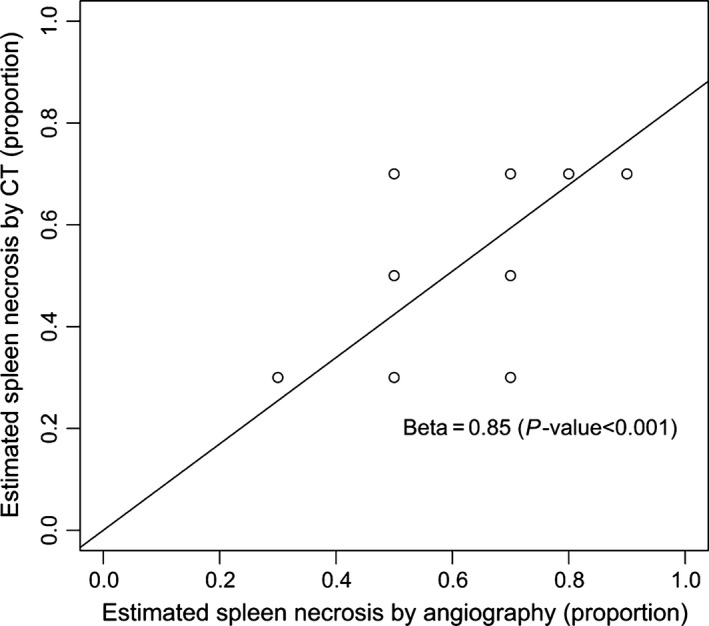
Estimated fraction of spleen necrosis by angiography significantly correlated with the estimated fraction of spleen necrosis by computed tomography.

Logistical and linear models did not show any association of number of cycles, estimated fraction of spleen necrosis by angiography, estimated fraction of spleen necrosis by CT, type of material, and size of particle with the percent of patients achieving platelet count >130 × 10^9^/L or with platelet count at week 2 (all *P* > 0.05). The estimated fraction of spleen necrosis by angiography significantly correlated with the estimated fraction of spleen necrosis by CT (Beta = 0.85, *P* < 0.001, linear regression), with angiography slightly overestimating the infarction area (Fig. 3).

## Discussion

In this study, we elected a platelet count of 130 × 10^9^/L as our primary endpoint since it is a safe value for the administration of SC. A total of 86% of the patients achieved platelets levels at that limit 2 weeks after PSE and, overall, it was reached by 94% of patients. These findings show how effective this approach can be which is also consistent with past studies 11, 12, 17. Recommence of anti‐cancer drugs, our secondary goal, occurred in all patients for whom SC was deemed necessary (in 31 out of 33 patients). Two patients did not return to SC, despite significant increase in the platelet count; the medical oncologist reached this decision after case review.

PSE is considered a safe procedure. Low‐grade fever, mild pain, and nausea are common and interpreted as a postembolization syndrome 11, 15, 18, 19. Even though severe complications, such as sepsis, portal vein thrombosis, overwhelming pulmonary infection and others, have been attributed to splenic embolization 2, 20, equivalent clinical worrisome conditions were not recognized in this study. One patient developed a single hematemesis episode with no serious consequences. Major complications after PSE usually arise from a large area of spleen embolization and are not necessarily associated with a more efficient result 17, 21, 22. In our study, most of the patients had a 50–70% infarction area with no major complications. In 60% of our cases, PSE was conducted as an outpatient procedure. Patients were kept overnight if they needed symptomatic treatment. This result is consistent with more recent publications in which overnight patient in‐hospital monitoring was not the rule 12, 23.

SC treatments, which involve oxaliplatin, have been linked to the appearance of distinctive liver lesions, such as hepatic sinusoids blockade, sclerosis of the perisinusoidal space, and veno‐occlusive disease, affecting the normal hepatic parenchyma and occasioning portal hypertension 14. Increases in spleen size, platelets splenic sequestration, and development of thrombocytopenia are surrogates of this toxicity 10. In our study, oxaliplatin‐based SC regimens were identified in 29 out of 33 patients (88%) before PSE and this could be one of the main reasons for the PSE effectiveness detected in which all patients demonstrated a significant and prompt increase in platelet count. Bevacizumab would be an interesting option in the treatment arsenal for this subset of patients since it has survival benefit in the metastatic colorectal malignancy but also because of its potential protective role against oxaliplatin‐mediated sinusoidal obstruction syndrome and splenomegaly as previously published 24. Unfortunately, this monoclonal humanized antibody directed against vascular endothelial growth factor is not routinely offered to patients with colorectal malignancy in our public cancer institution because of the high cost of this treatment in our country.

There was a statistically significant correlation of the estimated infarcted spleen area between angiography and CT post PSE (*P* < 0.001), with angiography slightly overestimating the area. Mean infarcted area estimation by angiography and CT was 59% and 43%, respectively. In one extreme case, the angiographic infarction appearance was 80% and at CT, it was 40%. In this particular case, the procedure time was very short (30 min) and the performing interventional radiologist might have rushed the procedure and not waited the recommended time for optimal embolization. The use of cone beam CT might be useful in achieving a more reliable analysis of the embolized spleen.

This study has important strengths and limitations that should be acknowledged. A strength of this study was the thorough evaluation of the primary cause of the decrease in the platelets count and its correlation with SC exposure 25, 26. Furthermore, very few studies have explored the actual role of PSE in the treatment of SC‐induced thrombocytopenia. We have currently addressed this issue as well as the fact that spleen embolization may indeed help patient's resumption of the anti‐cancer drug treatment 11, 12. As for limitations, these include being a single‐center study, no control group and short follow‐up time. Ideal criteria and perfect timing for the selection of patients should be well established to determine eligibility for the procedure. Perhaps, a control group aiming for a smaller spleen infarcted area to determine the best embolization fraction with focus in resumption of SC could be interesting. Overall survival in patients submitted to PSE to treat CIT could have been determined with a longer follow‐up.

In conclusion, PSE appears secure and efficient in handling thrombocytopenia secondary to SC facilitating the continuation of the proposed oncological regimen. The substantial and rapid expansion in the thrombocytes numbers following partial splenic embolization suggests that aiming for a no more than 70% infarcted area may be sufficient to achieve success without the complications associated with more extensive parenchymal infarction. This finding, combined with the better safety profile of PSE, makes it an excellent therapy option in the management of CIT, enabling patients to resume systemic chemotherapy with minimal procedure‐related morbidity.

## Conflict of Interest

The authors of this work declare no conflicts of interest.
